# Turning Cell Adhesions ON or OFF with High Spatiotemporal Precision Using the Green Light Responsive Protein CarH

**DOI:** 10.1002/chem.202001238

**Published:** 2020-06-03

**Authors:** Dongdong Xu, Julia Ricken, Seraphine V. Wegner

**Affiliations:** ^1^ Max Planck Institute for Polymer Research Ackermannweg 10 55128 Mainz Germany; ^2^ Max Planck Institute for Medical Research Jahnstraße 29 69120 Heidelberg Germany; ^3^ Institute of Physiological Chemistry and Pathobiochemistry University of Münster Waldeyerstraße 15 48149 Münster Germany

**Keywords:** CarH, cell adhesion, green light, photoswitchable, RGD

## Abstract

Spatiotemporal control of integrin‐mediated cell adhesions to extracellular matrix regulates cell behavior with has numerous implications for biotechnological applications. In this work, two approaches for regulating cell adhesions in space and time with high precision are reported, both of which utilize green light. In the first design, CarH, which is a tetramer in the dark, is used to mask cRGD adhesion‐peptides on a surface. Upon green light illumination, the CarH tetramer dissociates into its monomers, revealing the adhesion peptide so that cells can adhere. In the second design, the RGD motif is incorporated into the CarH protein tetramer such that cells can adhere to surfaces functionalized with this protein. The cell adhesions can be disrupted with green light, due to the disassembly of the CarH‐RGD protein. Both designs allow for photoregulation with noninvasive visible light and open new possibilities to investigate the dynamical regulation of cell adhesions in cell biology.

## Graphical abstract

**Spatiotemporal control** of cell adhesions with green light: Cell adhesions were triggered with green light using CarH as a nonadhesive photoresponsive layer to mask the cell adhesion peptide RGD. Contrariwise, the RGD peptide was incorporated into the CarH protein, such that cells can adhere and can be released upon green‐light exposure.

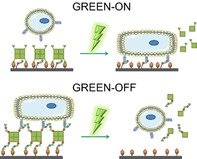

The cell adhesions to their extracellular matrix (ECM) are of fundamental importance in cell biology, and they are strictly regulated in space and time during different cellular processes including cell migration,^1^ differentiation,^2^ division^3^ and apoptosis.^4^ As such biomimetic synthetic materials have provided great insight into the mechanism surrounding cell adhesion processes and have guided material designs in medical applications, tissue engineering and cell‐based biosensing devices. Stimuli‐responsive ECM mimetic materials that allow altering cell adhesion in response to external stimuli, such as light,^5^ heat,^6^ pH^7^ and voltage,^8^ have attracted a lot of attention. Controlling cell adhesions with light is particularly attractive as it provides regulation with a subcellular spatial and high temporal resolution, unmatched by other stimuli. Studies using photocontrolled cell‐ECM interactions with high spatiotemporal control have provided new insight into the role of adhesions in intracellular signal transduction,^9^ dynamics of cell adhesions,^10^ mechanosensing,^11^ collective cell migration,^12^ vascularization of biomaterials,^13^ guiding neuronal growth^14^ and stem cell differentiation.^15^

Until now, numerous strategies to control cell‐ECM interactions with light have been developed. Towards this purpose, photocleavable groups (e.g., nitrobenzyl), which can be removed upon illumination with UV light, have been used to locally remove nonadhesive PEG (polyethylene glycol) chains and render the surface adhesive.^12^, ^16^ Likewise, photocleavable groups have been coupled to cell adhesion peptides such as the arginine‐glycine‐aspartic acid peptides (RGD), which are recognized by integrin receptors, as caging groups^13^, 15b or to release them.^17^ Linking these photocleavable groups to upconverting nanoparticles has allowed photoregulation with far‐red light, thereby overcoming the phototoxicity of UV‐light.^18^ For reversible on/off regulation, photoswitchable groups (e.g., azobenzene) have also been employed to regulate RGD accessibility for integrin binding and mechanical properties of hydrogels by using UV light.^19^ A recent advancement in the field that overcomes limitation in response to UV‐light is the integration of light responsive proteins into materials. These proteins respond to noninvasive visible light and can be genetically encoded so that cells can produce them. Hydrogel materials and surfaces coated with such light‐responsive proteins allow altering cell adhesion properties10c, 20 as well as mechanical properties^21^ and thereby, study the dynamics of associated cellular processes. Moreover, photoswitchable proteins have also been integrated into the integrin receptors^22^ and used as artificial cell adhesion receptors^23^ to photoregulate cell adhesions.

In this manuscript, we demonstrate how to control cell adhesions to RGD motives using the green‐light‐responsive protein, CarH, as a photosensitive unit. CarH from *Thermus thermophilus* forms a tetramer in the dark when it binds its cofactor AdoB_12_ and dissociates into monomers upon exposure to green light.^20^, ^24^ In two different designs, we were able to either turn cell adhesion on or off upon green‐light illumination.

In the first approach, GREEN‐ON, the tetrameric CarH protein was used to form a nonadhesive layer hiding an underlying cRGD adhesion peptides (Figure 1 a). Following green‐light illumination, the CarH protein layer was removed, and cells could adhere to the exposed cRGD sequences.

**Figure 1:**
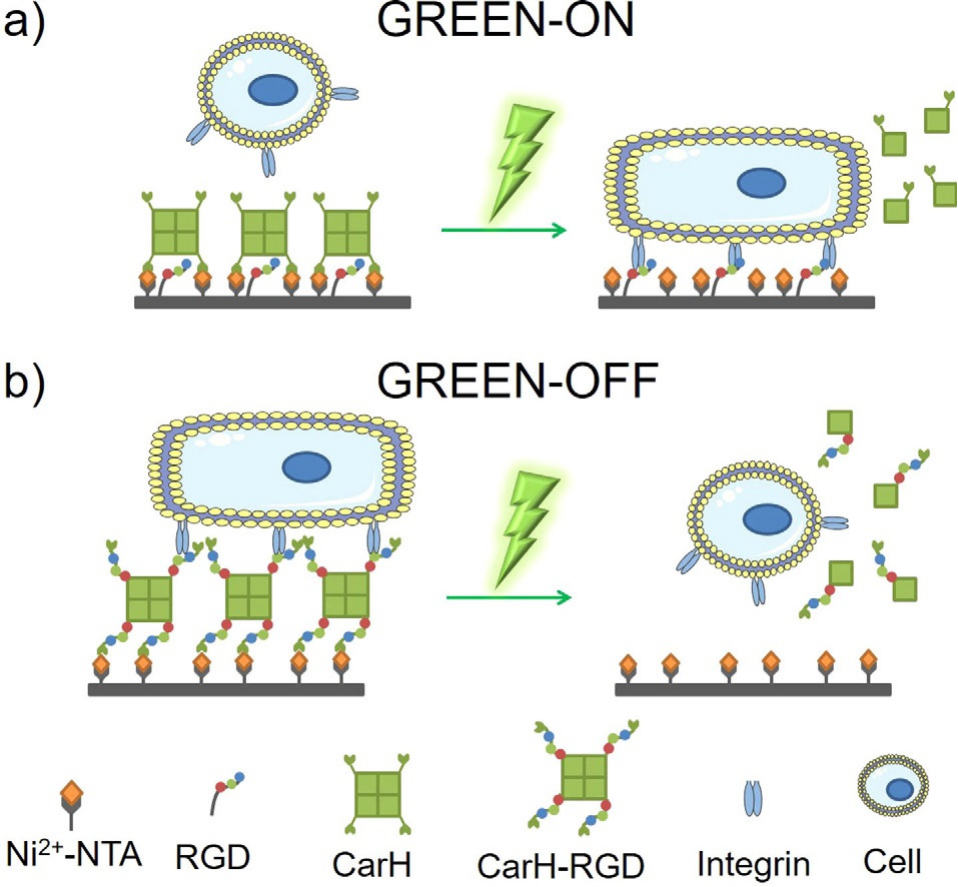
Green‐light‐controlled cell**‐**ECM adhesions. a) GREEN**‐**ON design: The CarH tetramer was employed as a nonadhesive layer, which masks the underlying cell adhesion peptide RGD and blocks the binding of integrins. The CarH was immobilized on a PEG coated surface through the NTA**‐**Ni^2+^/His**‐**tag binding. Upon green‐light illumination, CarH dissociates into its monomers and is removed from the surface, exposing the RGD peptides to integrin receptors on the cell and leads to cell adhesion. b) GREEN**‐**OFF design: The RGD adhesion motif was fused to the CarH to yield CarH**‐**RGD. A PEG coated surface was functionalized with the CarH**‐**RGD tetramer, so that the RGD motifs are exposed for cell adhesion in the dark. Under green light, CarH**‐**RGD dissociated and leads to the detachment of the cells from the surface.

In the second approach, GREEN‐OFF, we introduced three copies of the adhesion peptide RGD into the CarH protein, yielding CarH‐RGD (Figure S1). Thereby, cells could adhere to surfaces functionalized with this protein (Figure 1 b). However, when these surfaces were exposed to green light, the adhesive CarH‐RGD protein dissociated, and cells could no longer adhere to the PEG coating underneath. Overall, these two designs showed how biocompatible green light CarH could be employed as an alternative to photocleavable groups such as the UV‐sensitive nitrobenzyl group to photoregulate cell adhesions (Figure S2).

The two approaches were implemented as follows. In the GREEN‐ON design, the adhesion peptide cRGD was covered by a nonadhesive and photosensitive layer of CarH tetramer. For this purpose, glass surfaces were first coated with PEG‐azide (a PEG3000 with an azide and a triethoxy‐silane terminal group), and the azide groups at the surface were functionalized with a mixture of 0.2 % cRGD‐alkyne and 99.8 % NTA‐alkyne using the copper(I) catalyzed azide‐alkyne click reaction. Subsequently, the CarH tetramer (light‐sensitive C‐terminal adenosylcobalamin binding domain of CarH with a C‐terminal His6‐tag of *Thermus thermophilus*, referred to as CarH in the manuscript) was immobilized onto these surfaces using the specific binding of the His‐tags and the Ni^2+^‐NTA (nitrilotriacetic acid).^20^ We expected that upon green‐light illumination, the CarH tetramer would disassemble into its monomers and dissociate from the surface efficiently (12 % of the initial protein remained on the surface) as previously shown,^20^ so that the underlying cRGD motifs are exposed for cell adhesion. The efficient removal of CarH from the surface upon green‐light illumination is presumably due to the avidity of multiple His‐tags in the CarH tetramer, which is lost when it dissociates into its monomers.^20^

In the second design, GREEN‐OFF, the CarH‐RGD tetramer was immobilized on top of a PEG layer with Ni^2+^‐NTA end groups through the His‐tags of the protein. Similar to the parent CarH protein, CarH‐RGD forms a tetramer in the dark as shown by size exclusion chromatography (Figure S3) and undergoes the same photoreaction as shown by UV/Vis spectroscopy (Figure S4). Due to the geometry of the CarH‐RGD tetramer,24c the different C‐terminal poly‐RGD sequences and His‐tags point in opposite directions (Figure S1). Thus, we predicted that two of the poly‐RGD sequences close to the His‐tags implicated in immobilization would not be accessible to integrin binding as they will be covered by the protein, but the other two poly‐RGD sequences would be facing away from the surface and would be exposed for integrins to bind. Thus, upon green‐light exposure, the CarH‐RGD tetramer would disassemble, leaving no exposed adhesion motifs for cell adhesion, similar to the native proteins CarH.^20^

To investigate if cell adhesion can be altered based on these two approaches using green light, we incubated MCF‐7 cells on the above‐described surfaces. In these experiments, one set of surfaces was kept in the dark, and another set was exposed to green light for 5 minutes prior to cell seeding. A surface with Ni^2+^‐NTA‐PEG coating was used as a negative control. As measures of cell adhesion, the number of cells on the surfaces and their spreading area was determined based on the DAPI nuclei staining and phalloidin‐TRITC based actin cytoskeleton staining, respectively (Figures S5 and S6).

For the GREEN‐ON design, very few cells adhered to the surfaces functionalized with CarH that were kept in the dark; however, five times more cells adhered to the surfaces when exposed to green light (Figure 2 a). Moreover, cells cultured in the dark and green light showed a significant difference in their spreading area. Green‐light‐illuminated cells spread well and formed actin networks, but in the dark cells did not spread (Figure 2 b, Figure S5). Taken together, these findings validate the design principle and indicate that cRGD peptides were hidden under the CarH layer prior to the tetramer disassembly upon green‐light illumination.

**Figure 2:**
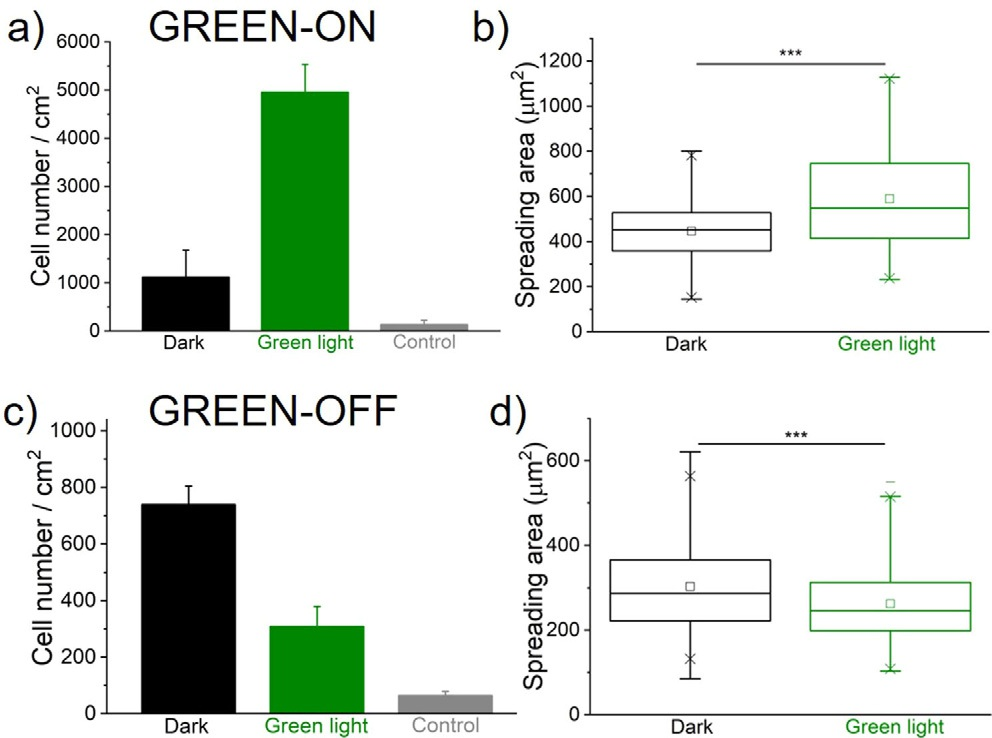
Cell adhesion on GREEN‐ON (a, b) and GREEN‐OFF (c, d) surfaces. Quantification of a), c) the number of cells that adhere based on the nuclear DAPI staining and b), d) spreading area of the cells based on the actin staining. The surfaces were either kept in the dark or illuminated with green light for 5 minutes prior to seeding cells. A surface without protein functionalization was used as a control. One Way ANOVA test, *p*‐value ***<0.001.

For the second strategy, GREEN‐OFF, where the MCF‐7 cells were seeded on CarH‐RGD functionalized surfaces, the opposite cell adhesion trends were observed. Cells adhered well to surfaces that were kept in the dark but adhered less to surfaces with prior green‐light exposure (Figure 2 c). Similarly, cells kept in the dark had a significantly larger spreading area on surfaces than ones exposed to green light (Figure 2 d, Figure S6).

Next, we explored the spatial and temporal control that these systems provide over cell adhesions in different set‐up. In a first example to demonstrate the spatial control, we used the GREEN‐ON system and projected a repeating green‐light stripe (150 μm) dark stripe (50 μm) pattern onto a surface. Then, we seeded MCF‐7 cells on the surface and stained the nuclei and actin cytoskeleton (Figure 3 a). The MCF‐7 cells adhered to the regions that were illuminated but not to the unilluminated regions reproducing the striped pattern.

**Figure 3:**
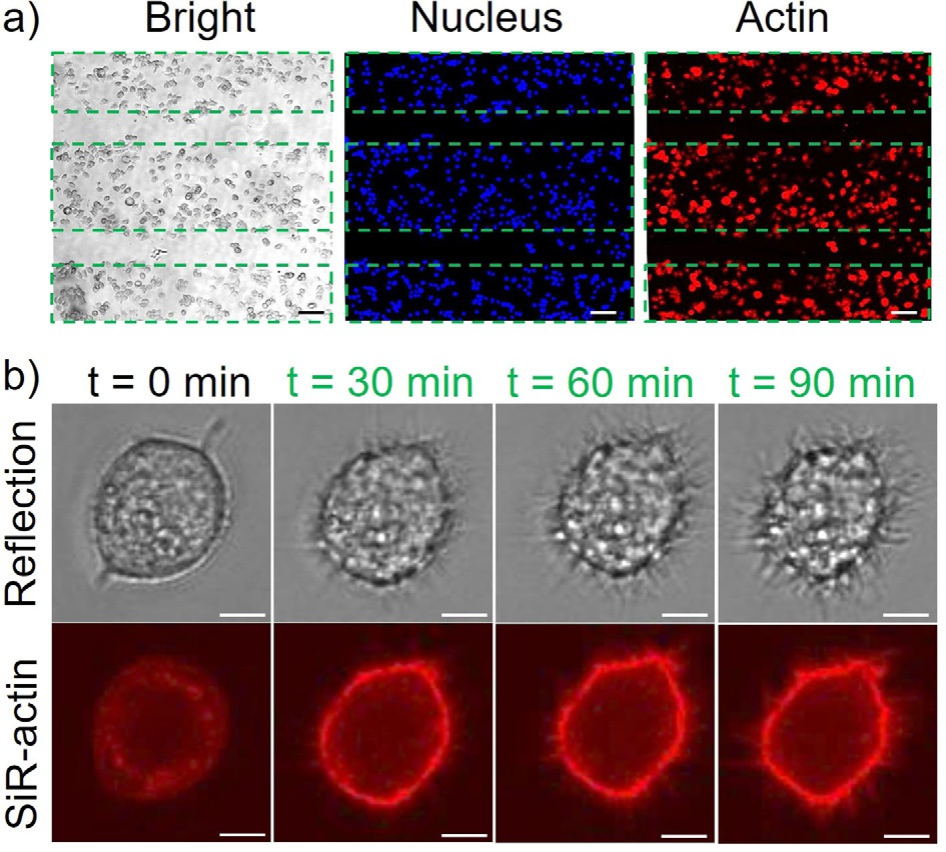
Spatial and temporal control over cell adhesions using the GREEN‐ON design. a) Patterned MCF‐7 cells seeded on surfaces that were pre‐exposed with a 150 μm wide pattern of green light (dash lines). Cell nuclei are shown in blue and the actin cytoskeleton in red. Scale bars are 50 μm. b) Green‐light‐triggered cell spreading. Top row: Interference reflection, bottom row: SiR‐actin staining. Scale bars are 5 μm.

In a second example the GREEN‐ON design was used to activate cell adhesions at any given time upon green‐light illumination, allowing to follow the progression of cell adhesions. To demonstrate this, cells, which were prestained with the SiR‐actin dye, were seeded onto the surface in the dark. The cells did not significantly adhere to the surfaces, as was evident by their rounded appearance and the lack of actin fibers. Subsequently, a cell that was sitting on the surface was illuminated with green light under a confocal laser scanning, and the changes in morphology and actin structures were monitored. Over the 90 min observation window, the cells spread and established focal contacts with the surface as both visible from the interference reflection channel and SiR‐actin actin staining (Figure 3 b and Figure S7).

Complementarily, the GREEN‐OFF design based on CarH‐RGD coated surfaces allows the disruption of cell adhesions when and where desired. The spatiotemporal control was demonstrated by allowing cells to adhere to a CarH‐RGD functionalized surface overnight (Figure 4). Then, in a field of view a subset of cells was chosen and the selected area was illuminated with green light under the microscope. We observed that the cells in the illuminated area detached from the surface within 20 min and the neighboring cells outside the illuminated area remained attached.

**Figure 4:**
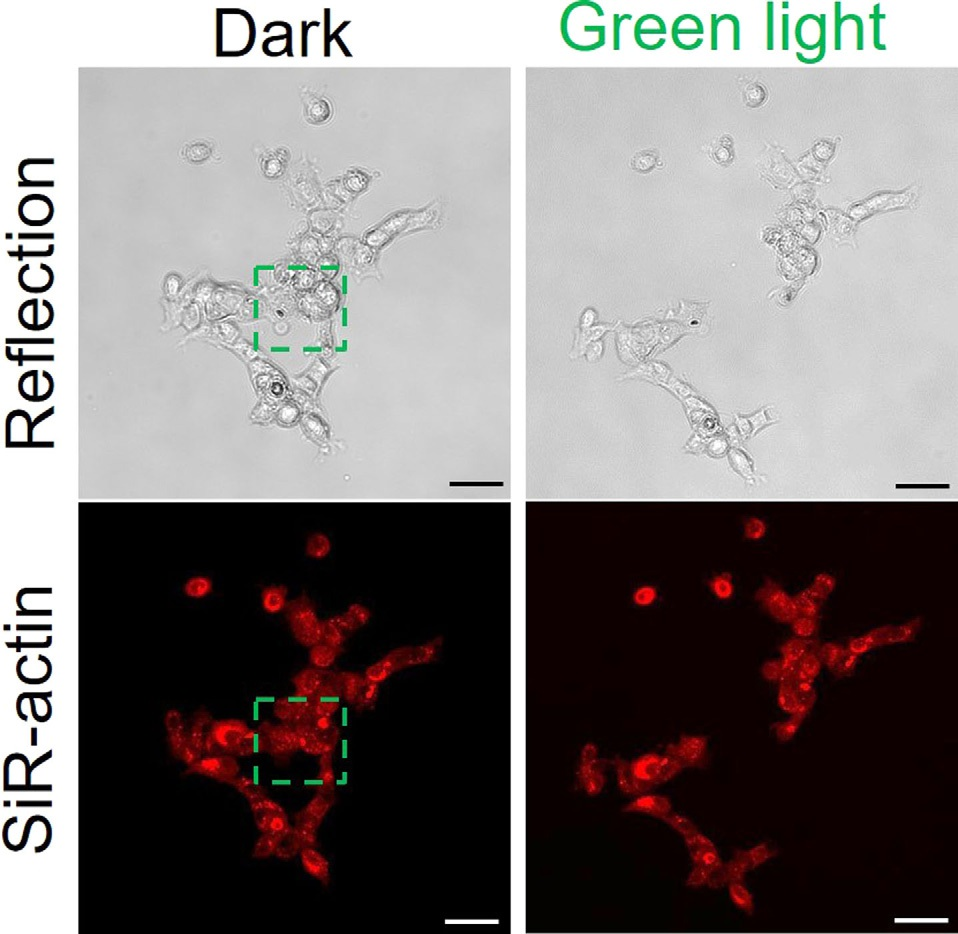
Spatial and temporal control over cell adhesions using the GREEN‐OFF design. Cells, which adhered to CarH‐RGD functionalized surfaces, were specifically removed in the green‐light‐illuminated area (green outline) over 20 min. Scale bars are 50 μm.

In conclusion, we have presented two approaches for green‐light‐regulated cell adhesions that provide noninvasive, biocompatable control over integrin mediated adhesions with high spatiotemporal precision. The photocleavable protein CarH, on which these approaches are based, provides a visible‐light‐responsive protein counterpart to the widely used UV‐light cleavable functional groups. In the GREEN‐ON approach, the CarH was used as a nonadhesive photocleavable layer to hide the adhesion peptide cRGD. This approach is very adaptable and CarH could be used to regulate the exposure of other adhesion or bioactive peptides as well as small molecules. Furthermore, CarH can also be incorporated as a light‐responsive alternative to non‐cell adhesive polymers, such as PEG, or proteins, such as BSA. In the GREEN‐OFF approach, we incorporated the RGD cell adhesion motif as a genetically coded protein component that can be removed with green light. Similarly, other peptide sequences or even protein domains could be incorporated as part of this green‐light‐removable protein. The high spatiotemporal precision and high flexibility, which these complementary GREEN‐ON and GREEN‐OFF designs provide, were here employed to grow cells in precise patterns, trigger cell spreading and remove a spatially defined subset of cells from a surface without the influence of phototoxic UV light. Yet, it should be noted that the response to low intensities of visible green light and the irreversible dissociation of the CarH tetramer requires handling and storing these surfaces carefully in the dark or under red light. Overall, the here presented design principles using CarH as a light‐responsive ECM component are an important step towards the biocompatible and spatiotemporal control of cell–matrix interactions. Given this, we anticipate that these strategies will be useful for the study of cellular events, such as differentiation, cell division, migration and metastasis, during which cell–ECM adhesions play an important role and for applications in tissue engineering and biomaterial development.

## Conflict of interest

The authors declare no conflict of interest.

## Biographical Information

*Seraphine Wegner obtained her Ph.D in Chemistry at the University of Chicago under the guidance of Prof. C. He in 2010. She was a postdoctoral researcher and later a group leader at the University of Heidelberg and Max Planck Institute for Intelligent Systems in the group of Prof. J. Spatz. In 2016, she became an independent group leader at the Max Planck Institute for Polymer Research as part of MaxSynBio. Since 2019, she is a full professor at Institute of Physiological Chemistry and Pathobiochemistry at the University of Münster. Her research focuses on the spatiotemporal control of cell‐material and cell–cell interactions and associated processes in natural and synthetic minimal cells by using visible light*.

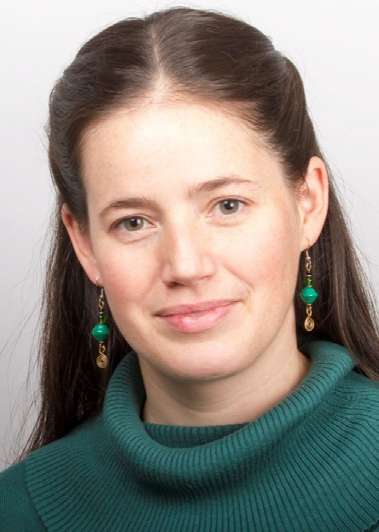

## Supporting information

As a service to our authors and readers, this journal provides supporting information supplied by the authors. Such materials are peer reviewed and may be re‐organized for online delivery, but are not copy‐edited or typeset. Technical support issues arising from supporting information (other than missing files) should be addressed to the authors.

Supplementary
