# Supplementary material for: Turning Cell Adhesions ON or OFF with High Spatiotemporal Precision Using the Green Light Responsive Protein CarH
Source: Chemistry. 2020 Jun 3;26(44):9859–63. doi: 10.1002/chem.202001238 (PMC7496717; doi:10.1002/chem.202001238)
Supplement: Supplementary file 1 — Supplementary [file CHEM-26-9859-s001.pdf]

Chemistry–A European Journal

Supporting Information

Turning Cell Adhesions ON or OFF with High Spatiotemporal Precision Using the Green Light Responsive Protein CarH

Dongdong Xu,[a] Julia Ricken,[a, b] and Seraphine V. Wegner*[a, c]

Support Information

Turning Cell Adhesions ON or OFF with High Spatiotemporal Precision using the Green Light Responsive Protein CarH

Dongdong Xu, Julia Ricken and Seraphine Wegner*

Experimental Procedures

Materials: All materials were commercially purchased and used without further purification. Buffers and aqueous solutions were prepared with Milli-Q water. The plasmid coding for the light sensitive C-terminal adenosylcobalamin binding domain of CarH with a C-terminal His6-tag in pet22b+ between the NdeI and XhoI cutting sites was a gift from Prof. Fei Sun.

Generation of CarH-RGD and purification of proteins: The three RGD motifs were inserted before the His-tag using the QuikChange II Site-Directed Mutagenesis Kit into the CarH plasmid.

Protein sequences:

```
CarH
MGELPEDLGTGLLEALLRGDLAGAEALFRRGLRFWGPEGVLEHLLLPVLREVGEAWHRGEIGVA
EEHLASTFLRARLQELLDLAGFPPGPPVLVTTPPGERHEIGAMLAAYHLRRKGVPALYLGPDTP
LPDLRALARRLGAGAVVLSAVLSEPLRALPDGALKDLAPRVFLGGQGAGPEEARRLGAEYMEDL
KGLAEALWLPRGPEKEAIGSGLEHHHHHH
```

```
CarH-RGD
MGELPEDLGTGLLEALLRGDLAGAEALFRRGLRFWGPEGVLEHLLLPVLREVGEAWHRGEIGVA

EEHLASTFLRARLQELLDLAGFPPGPPVLVTTPPGERHEIGAMLAAYHLRRKGVPALYLGPDTP

LPDLRALARRLGAGAVVLSAVLSEPLRALPDGALKDLAPRVFLGGQGAGPEEARRLGAEYMEDL

KGLAEALWLPRGPEKEAIGSGLQRGDRGDRGDRGDHHHHHH
```

For protein expression and purification, the plasmids coding for CarH or CarH-RGD were first transformed into BL21 (DE3) *E. coli* (New England Biolabs). Then, an overnight culture was started from a single colony in 10 mL LB medium with 50 µg/mL ampicillin at 37 ºC, 150 rpm. The overnight culture was transferred into 1 L LB medium with 50 µg/mL ampicillin, incubated at 37 ºC, 200 rpm until the OD_{600} = 0.4-0.6 and then the protein expression was induced with 500 µM IPTG (isopropyl β-D-1-thiogalactopyranoside). The cultures were incubated at 16 ºC, 200

rpm overnight and harvested the next day by centrifugation at 6000 rpm, 4 ºC for 8 min (Beckman Coulter Avanti J-26S XP, JA-10 rotor). The bacteria pellet was resuspended in 20 mL buffer A (300 mM NaCl, 50 mM Tris, pH = 7.4) supplemented with 1 mM protease inhibitor phenylmethane sulfonyl fluoride (PMSF) and 1 mM DL-dithiothreitol (DTT). The bacteria were lysed by sonication and the lysate was cleared by centrifugation at 12000 rpm (Beckman Coulter Avanti J-26S XP, JA-25.50 rotor) for 30 min, followed by filtration through a 0.45 µm filter twice. The lysate was loaded onto a 5 mL Ni^{2+}-NTA agarose column (HisTrap™ HP, column volume 5 mL). The column was washed with 50 mL Buffer A with 25 mM imidazole and 1 mM DTT and the protein was eluted with 10 mL Buffer A with 250 mM imidazole and 1 mM DTT. The purified proteins were dialyzed against 2 L Buffer A with 1 mM DTT twice for at least 6 h at 4 ºC. The CarH-RGD tetramer complex was assembled upon addition of a four-fold molar excess of $AdoB_{12}$ to the monomer solution in the dark and incubation at 4 °C overnight. The obtained tetramer was further purified utilizing a size exclusion column (GE Healthcare, HiLoad 16/600, Superdex 200 pg) with Buffer A (Figure S3). The photoresponse of the CarH-RGD tetramer upon green light illumination (green LED, 515 nm – 520 nm) was followed with UV-Vis spectroscopy (Tecan infinite M200, Tecan, Männedorf, Switzerland) over 1 min (Figure S4).

Preparation of protein functionalized glass surface: Glass slides (20 × 20 mm) were cleaned with freshly prepared Piranha solution (3:1 (v/v) concentrated H_2SO_4:H_2O_2 (30%)) for 1 h, rinsed 3 times with Milli-Q water and dried in an N_2 stream. For the PEGylation reaction, surfaces were immersed in a solution of PEG_{3000}-azide (10 mg PEG_{3000}-azide, MW = 3500 g/mol) and 200 µl dry triethylamine in dry toluene and kept at 80 ºC overnight under a N_2 atmosphere. The surfaces were first washed with ethyl acetate for 5 min by sonication, then with methanol for 5 min by sonication and dried in a N_2 stream. Each PEG-azide coated surface was incubated with 100 µl of reaction solution containing 100 mM L-ascorbic acid, 100 mM Tris HCl (pH 9.0), 150 µM of NTA-alkyne, (0.3 µM of cRGD-alkyne was added additionally for the GREEN-ON design) and 1 mM $CuSO_4$ in a moisture chamber for 2 h. The surfaces were incubated with the following solutions to obtain PEG-Ni^{2+}-NTA functionalities on the surfaces: (1) 50 mM EDTA (pH 7.4) for 5 min; (2) Buffer A (50 mM Tris-HCl, 300 mM NaCl, pH 7.4) twice for 5 min; (3) 0.1 M $NiCl_2$ in water for 5 min; (4) Buffer A twice for 5 min.

Afterward the surfaces were incubated first with 5 µM purified protein for 30 min and the washed with Buffer A for 10 min. Surfaces for the GREEN-ON and GREEN-OFF designs CarH and CarH-RGD were used as proteins, respectively.

Quantification of cell adhesion: One set of surfaces functionalized with CarH and cRDG (GREEN-ON) or CarH-RGD (GREEN-OFF) was illuminated with green light for 5 min (15 W), the other set was kept in the dark. A PEG-Ni^{2+}-NTA functionalized surface without protein functionalization was used as a negative control. The surfaces were placed into the 6-well cell culture plates and were washed with phosphate buffered saline 1x (PBS). Subsequently, 2 x 10^5 MCF-7 cells were seeded per well in 2 ml Dulbecco's modified Eagle's medium (DMEM) (Gibco BRL) supplemented with 10% heat inactivated fetal bovine serum (FBS) (Sigma Aldrich) and 1% penicillin/streptomycin (P/S) (Gibco BRL) and incubated at 37 ºC, 5% CO_2 in the dark. For the GREEN-ON and the GREEN-OFF design the cells were incubated on the surfaces overnight and for 2 hours, respectively. Subsequently, the surfaces were washed twice with PBS and the cells were fixed with 2% paraformaldehyde at room temperature for 20 min. After fixation, the surfaces were washed twice with PBS, permeabilized with 0.1% v/v Triton X-100 in PBS at room temperature for 5 min and stained with 0.5 µg/mL phalloidin-TRITC in PBS at room temperature for 30 min for actin staining. The cells were then mounted with Moviol-488 containing 1 µg/ml DAPI (Sigma Aldrich) for nucleus staining. Fluorescent images were acquired with an inverted fluorescence microscope (DMi8, Leica) in the DAPI and TRITC channel with a 10x objective for an area of 5 mm^2 per sample. The number of cells was quantified using the DAPI staining and the cell spreading area was the analyzed based on the actin staining (100 single cells were analyzed per condition) using Fiji ImageJ. Each experiment was performed in biological triplicates. In the box plots, the box represents the 25–75% percentile and the whiskers are the 10–90% percentiles. Statistical significance is evaluated using the One Way ANOVA test. *p*-value *** <0.001.

Metabolic Activity Test: The influence of light on the metabolic activity of cells was determined with the 3-(4,5-Dimethylthiozol-2-yl)-2,5-diphenaltetrazolium bromide (MTT) assay. Therefore, MCF-7 cells were seeded in a density of 50000 cells/cm^2 in a 96 well plate in 100 µL complete growth medium (DMEM + 10% FBS + 1% P/S). The cells were incubated at 37 °C exposed to green light (532 nm, Flora LED Module) at different intensities for 2 h. The control was kept meanwhile in the dark. Afterwards 75 µL medium per well were removed and 10 µL MTT (saturated solution in PBS) was added. The cells were incubated at 37 °C in dark for 4 h. 50 µL DMSO per well was added and incubated at room temperature in dark for 10 min. The absorption at 540 nm was measured with a Tecan plate reader. The lowest tested light intensity at 12.5 µM/m^2s was used in all cell experiments, to assure no influence of the cells.

Cell spatial and temporal control of cell adhesions: To pattern cells on GREEN-ON surfaces, surfaces modified with CarH and cRGD-alkyne peptide were illuminated in a desired pattern using the FRAP function with green light at 552 nm (argon laser, 10% intensity) for 1 min under a confocal laser scanning microscopy (Leica TCS SP8) through a 10x objective. Then, the surfaces were incubated with 1×10^6 MCF-7 cells in the 2 ml DMEM supplemented with 10% FBS and 1% P/S and 300 nM SiR-actin for actin staining at 37 ºC, 5% CO_2 overnight. Subsequently, 1 µg/ml Hoechest 33342 was added to stain the cell nuclei for 10 min and surfaces were washed with PBS before acquiring images of the cells on the surfaces in the actin and nuclear stain channels.

To trigger cell adhesions or detach cells upon green light illumination, CarH/cRGD-alkyne (GREEN-ON) or CarH-RGD (GREEN-OFF) functionalized glass surfaces were glued to a hollow µ-slide well dish (ibidi). For the GREEN-ON surfaces 2×10^5 MCF-7 cells and for the GREEN-OFF surfaces 1×10^5 MDA-MB-231 cells were seeded in the 2 ml DMEM supplemented with 10% FBS and 1% P/S containing and 300 nM SiR-actin for actin staining and incubated at 37 ºC, 5% CO_2 overnight. The surfaces were washed twice with PBS and fresh DMEM supplemented with 10% FBS and 1% P/S was added. The cells were imaged in the interference reflection and SiR channels. For the cells on the GREEN-ON surface, single cells with lose initial contacts to the surface were identified and illuminated with green light at 552 nm (1% intensity) for 30 min using the FRAP function and the cells were imaged over the course of 90 min. For the GREEN-OFF surfaces, a group of adhered cells was places in the field of view and a subset of cells in a chosen area were illuminated with green light at 552 nm (1% intensity) for 20 min using the FRAP function. The surface was once washed with PBS and images in the different channels were acquired.

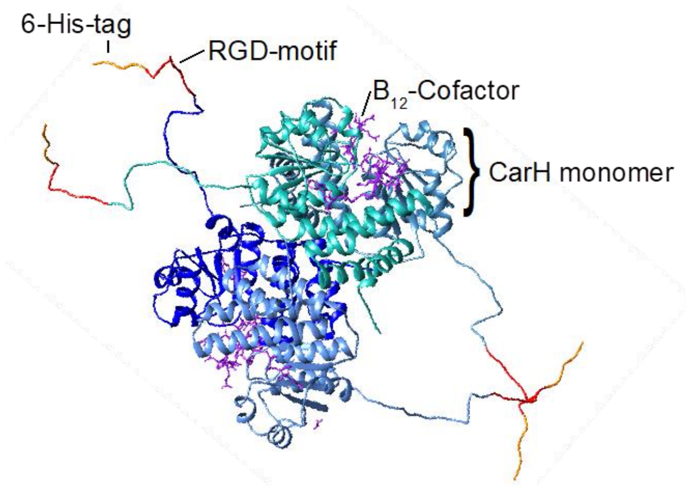

Figure S1: Design of CarH-RGD. a) Cobalamin binding domain of CarH in the dark is a tetramer (PDB: 5C8A) shown in different tones of blue and the cofactor $AdoB_{12}$ in purple. The predicted position of the C-terminal RGD motifs and His-tag are shown in red and yellow, respectively.

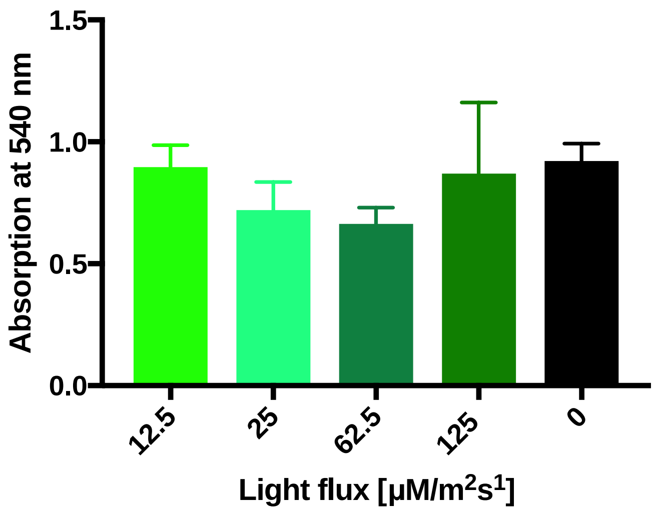

Figure S2: The metabolic activity of MCF-7 cells after green light illumination for 2 hours and in the dark, was assessed with a MTT assay, showing no decreased activity of cell cultures under different light intensities of green light compared to dark, which shows the nontoxicity of green light.

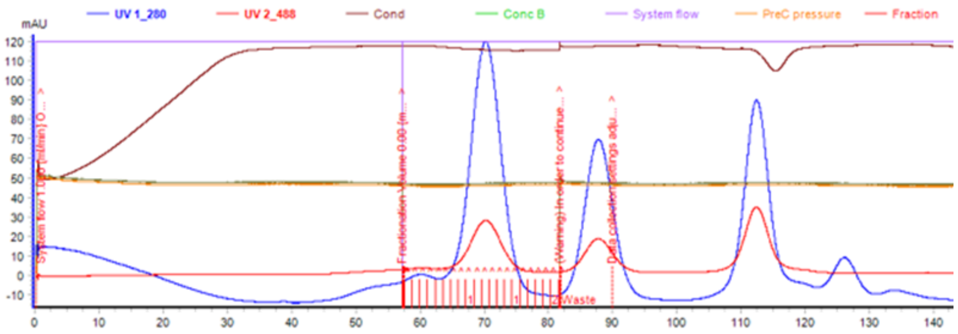

Figure S3: Chromatogram of CarH-RGD on a size exclusion column. Blue curve: Absorbance at 280 nm, red curve: absorbance at 488 nm. The CarH-RGD tetramer eluted at 70 mL, the CarH-RGD monomer at 87 mL and the excess cofactor $AdoB_{12}$ at 115 mL.

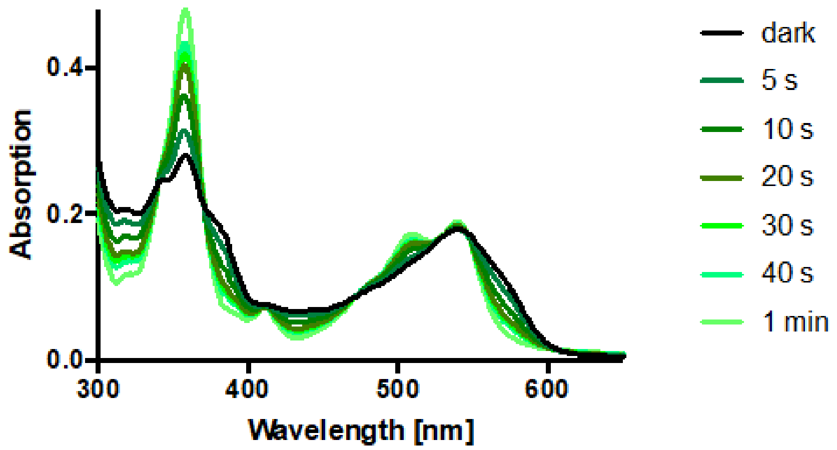

Figure S4: UV-Vis spectra of the CarH-RGD tetramer upon disassembly under green light. The black curve presents CarH tetramer before exposure to green light and the green curves show the changes over time under green light illumination.

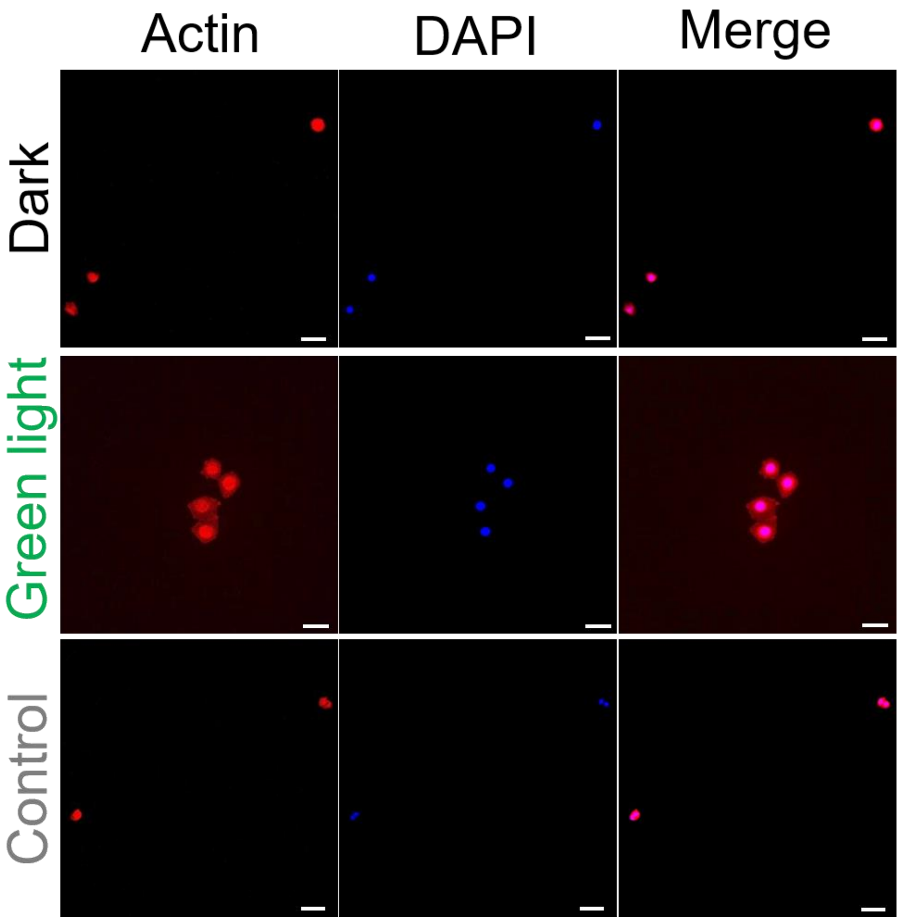

Figure S5: Fluorescence images of cells on GREEN-ON surfaces in the dark and under green light illumination. A surface without any protein and peptide functionalization was used as a negative control. Red: actin, blue: nucleus. Scale bar: 50 µm.

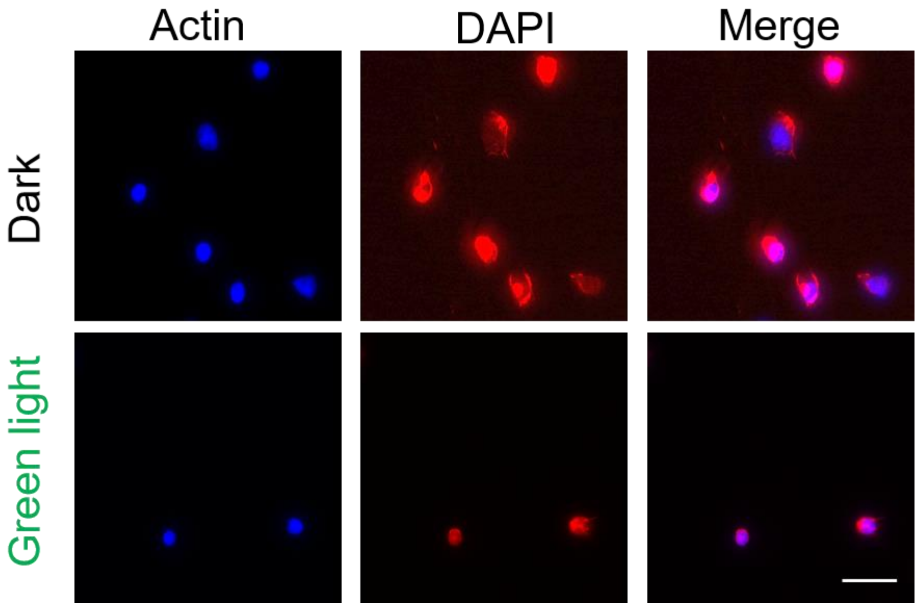

Figure S6: Fluorescence images of cells on GREEN-OFF surfaces in the dark and under green light illumination. In the merged channel, red: actin, blue: nucleus. Scale bar: 50 μm.

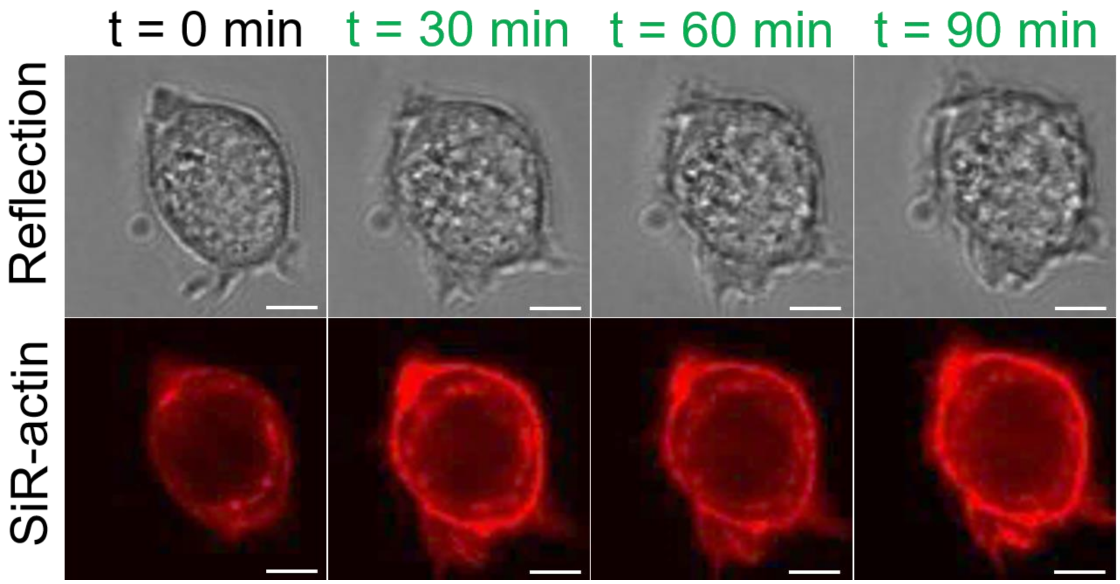

Figure S7: Cell spreading on the GREEN-ON surfaces under green light illumination as monitored with live cell imaging in the interference reflection and SiR-actin channels. Images were first acquired in the dark (t = 0 min) and the cell spreading was monitored over time after 30 min green light illumination. Scale bar: 5 μm.
